# L1CAM/CD171 expression in human tumors and its association with tumor phenotype

**DOI:** 10.2340/1651-226X.2025.43587

**Published:** 2025-12-02

**Authors:** Seyma Büyücek, Magalie Lurati, Katharina Möller, Florian Viehweger, Ria Schlichter, Anne Menz, Andreas M. Luebke, Viktor Reiswich, Martina Kluth, Claudia Hube-Magg, Andrea Hinsch, Florian Lutz, Sören Weidemann, Frank Jacobsen, David Dum, Christian Bernreuther, Patrick Lebok, Guido Sauter, Andreas H. Marx, Ronald Simon, Christoph Fraune, Natalia Gorbokon, Eike Burandt, Sarah Minner, Stefan Steurer, Till S. Clauditz, Till Krech, Viktoria Chirico, Maximilian Lennartz

**Affiliations:** a Institute of Pathology, University Medical Center Hamburg-Eppendorf, Hamburg, Germany; b Institute of Pathology, Clinical Center Osnabrueck, Osnabrueck, Germany; c Department of Pathology, Academic Hospital Fuerth, Fuerth, Germany

**Keywords:** L1CAM, cell adhesion molecules, biomarkers, tumor, membrane proteins, tissue array analysis, immunohistochemistry

## Abstract

**Background and purpose:**

L1CAM (CD171) is suggested to play a critical role in cancer. Because of its expression in only few normal tissues and its membranous nature, L1CAM is a promising drug target.

**Patient/material and methods:**

To clarify the role of L1CAM expression in different cancer types, a tissue microarray containing 20,079 samples from 135 different tumor entities and 608 samples of 76 different normal tissue types was analyzed by immunohistochemistry.

**Results:**

Membranous L1CAM staining was found in 1,175 (9.1%) of 12,888 interpretable tumor samples, including 301 (2.3%) with weak, 569 (4.4%) with moderate, and 305 (2.4%) with strong positivity. 74 of 135 tumor entities showed L1CAM staining, and 36 tumor categories included at least one case with strong L1CAM staining. The frequency of L1CAM positivity was high in subtypes of neural and neuroendocrine neoplasms (up to 100%), endometrium carcinoma (24.1-31.3%), ovarian cancer (10.0-33.1%), cervical adenocarcinoma (9.1%), malignant melanoma (24.1-31.3%), malignant mesothelioma (16.7-20.8%), adenocarcinomas of the gastrointestinal and biliopancreatic tract (4.9-14.1%), and in urothelial tumors (up to 10.3%). High L1CAM expression was associated with invasive tumor growth (pTa vs. pT2-4) in urothelial carcinoma of the bladder (p<0.0001) and with mismatch repair deficiency in colorectal adenocarcinoma (p=0.0064). However, L1CAM staining was unrelated to tumor phenotype in seven other tumor entities.

**Interpretation:**

The results highlighted a small number of tumor entities that could be targeted by anti-L1CAM drugs, once these are proved to be sufficiently safe and efficient. L1CAM expression does not appear to confer an aggressive phenotype to affected cancer cells.

## Introduction

The L1 cell adhesion molecule (CD171; L1CAM) is a transmembrane glycoprotein of the immunoglobulin superfamily which was initially characterized as a cell surface antigen of the central nervous system [1, 2]. L1CAM plays an important role in the regulation of neuronal cell migration, adhesion, neuronal differentiation, myelination and axon growth (summarized in [3]). Germline L1CAM mutations cause severe disorders of brain development, including corpus callosum hypoplasia, retardation, aphasia, spastic paraplegia and hydrocephalus (CRASH syndrome) [4]. In normal tissues, L1CAM expression occurs in only a few non-neural cell types. However, L1CAM protein can be aberrantly expressed in various cancer types [5, 6].

Data from various cancer studies have linked L1CAM protein expression to tumor progression [7], metastasis [8], therapy resistance [9], stemness [10], and dismal prognosis [7, 11] (summarized in [12]). Evidence for a prognostic role of aberrant L1CAM expression has been found in endometrial cancer [13], oral squamous cell carcinoma [14], carcinosarcoma of the uterus [15], cervical cancer [16], serous carcinoma of the ovary [17], adenocarcinoma of the lung [18], ductal adenocarcinoma of the pancreas [19], esophageal squamous cell carcinoma [20], colorectal cancer [21] and neuroblastoma [22]. The potential role of L1CAM in tumor progression, in combination with its membranous location and its expression in only a few normal cell types, makes L1CAM an attractive candidate drug target. Targeting L1CAM protein in cancer cells is currently being evaluated by various approaches, including neutralizing antibodies [23], bispecific antibodies [24], radioimmunoconjugates [25] and chimeric antigen receptor-redirected T (CAR-T) cells [26]. More than 200 studies have so far analyzed L1CAM expression in cancer by immunohistochemistry (IHC). Although many of these studies have suggested associations between L1CAM expression and increased cancer aggressiveness, the available data on the prevalence of L1CAM expression are remarkably discrepant. For example, L1CAM positivity ranged from 2 to 100% in neurofibroma, 24–95% in serous carcinoma of the ovary, 24–96% in malignant melanoma, 0–56% in colorectal adenocarcinoma, 3–100% in endometrial cancer, 21–100% in papillary renal cell carcinoma (RCC), 2–93% in ductal adenocarcinoma of the pancreas, 11–63% in hepatocellular carcinoma and 2–74% in gastrointestinal stromal tumor (GIST) (Table 3). These conflicting data may be caused by the use of different antibodies, immunostaining protocols and criteria to define L1CAM positivity in these studies.

To better understand the prevalence and clinical significance of L1CAM expression in cancer, a comprehensive study analyzing a large number of neoplastic and non-neoplastic tissues under highly standardized conditions is needed. Therefore, L1CAM expression was analyzed in more than 20,000 tumor tissue samples from 135 different tumor types and subtypes, as well as 76 non-neoplastic tissue categories, using IHC in a tissue microarray (TMA) format in this study.

## Patients/material and methods

### Tissue microarrays

Our normal TMA was composed of samples from 8 different donors from each of 76 different normal tissue categories (608 samples on one slide). The cancer TMAs included a total of 20,079 primary tumors from 135 different tumor types and subtypes. Detailed histopathological and molecular data were available for cancers of the bladder (*n* = 2,434), colon (*n* = 2,351), kidney (*n* = 1,757), ovary (serous high-grade; *n* = 369), endometrium (endometrioid; *n* = 182), stomach (*n* = 327), and the pancreas (*n* = 598). The composition of normal and cancer TMAs is described in the results section. All samples were obtained from the archives of the Institute of Pathology, University Medical Center Hamburg-Eppendorf, Germany, the Institute of Pathology, Clinical Center Osnabrueck, Germany and the Department of Pathology, Academic Hospital Fuerth, Germany. Tissues were fixed in 4% buffered formalin and then embedded in paraffin. The TMA manufacturing process has been described earlier in detail [27, 28]. In brief, one tissue spot (diameter: 0.6 mm) per patient was used. The use of archived remnants of diagnostic tissues for TMA manufacturing, their analysis for research purposes, and the use of patient data were according to local laws (HmbKHG, §12) and data analysis had been approved by the local ethics committee (Ethics commission Hamburg, WF-049/09). All work has been carried out in compliance with the Helsinki Declaration.

### Immunohistochemistry

Freshly cut TMA sections were stained on one day and in one experiment. Slides were deparaffinized with xylol, rehydrated through a graded alcohol series and exposed to heat-induced antigen retrieval for 5 minutes in an autoclave at 121°C in pH 7.8 Tris-EDTA-Citrat (TEC) buffer. Endogenous peroxidase activity was blocked with Dako REAL Peroxidase-Blocking Solution (Agilent Technologies, Santa Clara, CA, USA; #S2023) for 10 minutes. The primary antibody specific for L1CAM (recombinant rabbit monoclonal, MSVA-171R, MS Validated Antibodies GmbH, Hamburg, Germany, #6263-171R) was applied at 37°C for 60 minutes at a dilution of 1:100. For the purpose of antibody validation, the normal tissue TMA was also analyzed by the mouse monoclonal L1CAM antibody 14.10 (BioLegend, San Diego, CA, USA; #826701) at a dilution of 1:50 and an otherwise identical protocol. The bound antibody was then visualized using the Dako REAL EnVision Detection System Peroxidase/DAB+, Rabbit/Mouse kit (Agilent Technologies, Santa Clara, CA, USA; #K5007) according to the manufacturer’s instructions. The sections were counterstained with hemalaun. For tumor tissues, the percentage of positive neoplastic cells was estimated, and the staining intensity was semi-quantitatively recorded (0, 1+, 2+, 3+). For statistical analyses, the staining results were categorized into four groups. Tumors without any staining and positive staining in less than 10% of tumor cells were considered negative. Tumors with 1+ staining intensity in ≤ 70% of tumor cells or 2+ intensity in ≤ 30% of tumor cells were considered weakly positive. Tumors with 1+ staining intensity in > 70% of tumor cells, 2+ intensity in 31–70%, or 3+ intensity in ≤ 30% of tumor cells were considered moderately positive. Tumors with 2+ intensity in > 70% or 3+ intensity in > 30% of tumor cells were considered strongly positive.

### Statistics

Statistical calculations were performed with JMP17® software (SAS®, Cary, NC, USA). Contingency tables and the chi²-test were performed to search for associations between L1CAM immunostaining and tumor phenotype.

## Results

### Technical issues

A total of 12,888 (64.2%) of 20,079 tumor samples were interpretable in our TMA analysis. Non-interpretable samples demonstrated lack of unequivocal tumor cells or lack of entire tissue spots. A sufficient number of samples (≥ 4) of each normal tissue type was evaluable.

### L1CAM in normal tissues

**Figure 1:**
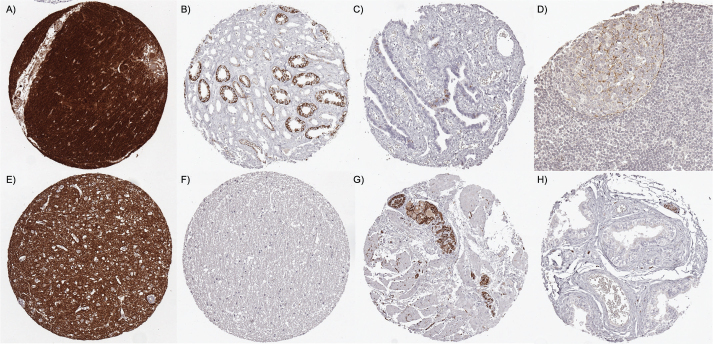
L1CAM immunostaining of normal tissues. The panel shows intense staining in the cerebellar cortex (A), strong predominantly membranous staining in a subset of collecting duct cells of the kidney medulla (B), strong staining of a small subset of epithelial cells of fallopian tube (C), weak to moderate staining of subsets of B-cells and monocytic cells, mainly of the germinal center of lymph node (D), intense staining in the grey matter of the cerebrum (E), lack of staining of the white matter of the cerebrum (F), strong staining of nerve fibers of the muscular wall of the esophagus (G) and lack of staining in the epididymis (staining only occurs in a nerve) (H). L1CAM: L1 cell adhesion molecule.

L1CAM staining was most intense in nerve fibers, especially in the grey matter of the cerebrum, the molecular layer of the cerebellum and the neurohypophysis, but also in many other organs, such as for example the muscular wall and the mucosa of the gastrointestinal tract, as well as in the stroma of the prostate and the seminal vesicles. A strong membranous L1CAM staining also occurred in medullary cells of the adrenal gland, a fraction of collecting ducts of the kidney, a small fraction of epithelial cells of the fallopian tube and – less intense – in chorion cells of the placenta and a subset of epithelial cells in the adenohypophysis. A weak to moderate L1CAM staining was also seen in subsets of B-cells and monocytic cells, mainly in the germinal centers of lymphatic tissues. Very rarely, L1CAM staining was also observed in endothelial cells. Representative images are shown in Figure 1. All these cell types identified by MSVA-171R were confirmed by clone 14.10 on consecutive tissue sections (Supplementary Figure 1). Except for nerve fibers, L1CAM staining was absent in thyroid, parathyroid gland, respiratory epithelium, lung, salivary glands, esophagus, liver, gallbladder, pancreas, urothelium, testis, epididymis, breast, myometrium of the uterus, ectocervix, endocervix, and endometrium of the uterus, ovary, first trimester and mature placenta, amnion, epidermis, sebaceous glands, skeletal heart, and smooth muscle, vessel walls, fat, bone marrow, spleen and the thymus.

### L1CAM in tumor tissues

**Figure 2:**
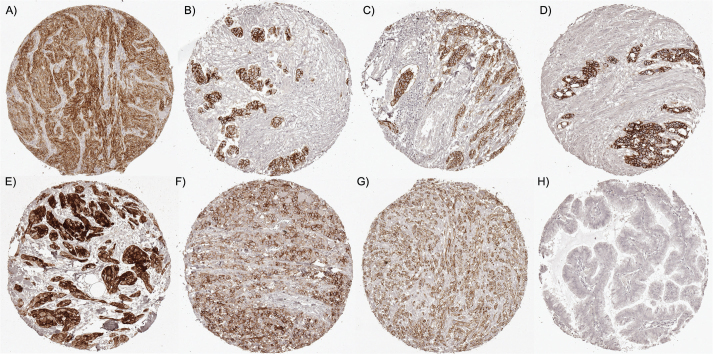
L1CAM immunostaining in cancer. The panel shows an intense, predominantly membranous L1CAM immunostaining in a neuroendocrine tumor of esophagus (A), a serous high-grade ovarian carcinoma (B), an invasive urothelial carcinoma (C), a gastric adenocarcinoma (D), a granular cell tumor (E), a colorectal adenocarcinoma (F) and a melanoma (G). L1CAM staining is lacking in a pulmonary adenocarcinoma (H). L1CAM: L1 cell adhesion molecule.

**Figure 3:**
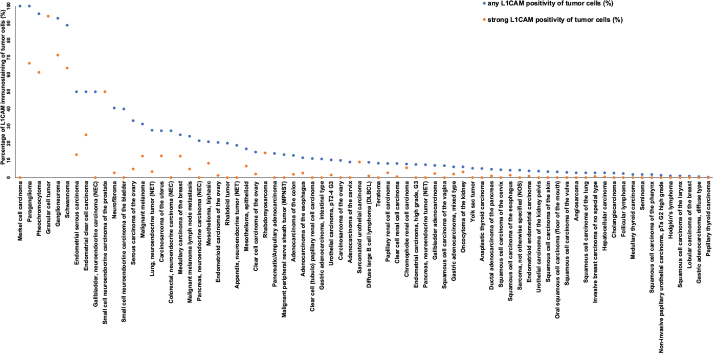
Ranking order of L1CAM immunostaining in tumors. Both the percentage of positive cases (blue dots) and the percentage of strongly positive cases (orange dots) are shown. L1CAM: L1 cell adhesion molecule.

A predominantly membranous L1CAM staining was found in 1,175 (9.1%) of the 12,888 interpretable tumor samples, including 301 (2.3%) with weak, 569 (4.4%) with moderate, and 305 (2.4%) with strong positivity. A total of 74 of 135 tumor entities (54.8%) showed L1CAM staining in at least one case, and 36 tumor categories (26.7%) included at least one case with strong L1CAM staining (Table 1). The frequency of L1CAM positivity was particularly high in several subtypes of neural and neuroendocrine neoplasms (up to 100%), endometrium carcinoma (3.9–50.0%), ovarian cancer (10.0–33.1%), cervical adenocarcinoma (9.1%), malignant melanoma (24.1–31.3%), malignant mesothelioma (16.7–20.8%), adenocarcinomas of the gastrointestinal and the biliopancreatic tract (4.9–14.1%) and in urothelial tumors (up to 10.3%). Representative images are shown in Figure 2. A graphical representation of a ranking order of L1CAM positive cancers and of strongly positive cancers is given in Figure 3. The relationship between L1CAM immunostaining and parameters of cancer aggressiveness is summarized in Table 2. High L1CAM expression was associated with invasive tumor growth (pTa vs. pT2–4) in urothelial carcinoma of the bladder (*p* < 0.0001) and with mismatch repair protein deficiency (*p* = 0.0064) and left-sided tumor location (*p* = 0.0104) in colorectal adenocarcinoma. L1CAM staining was unrelated to tumor phenotype in muscle-invasive urothelial carcinoma, clear cell and papillary RCC, serous high-grade carcinoma of the ovary, endometrioid endometrium carcinoma, gastric adenocarcinoma and in pancreatic ductal adenocarcinoma.

**Table 1:** L1CAM immunostaining in human tumors.

	Tumor entity	on TMA (n)	L1CAM immunostaining				
			analyzable (n)	negative (%)	weak (%)	moderate (%)	strong (%)
Tumors of the skin	Pilomatricoma	35	35	100,0	0,0	0,0	0,0
	Basal cell carcinoma of the skin	89	35	100,0	0,0	0,0	0,0
	Squamous cell carcinoma of the skin	145	87	96,6	0,0	3,4	0,0
	Malignant melanoma	65	16	68,8	0,0	18,8	12,5
	Malignant melanoma lymph node metastasis	86	79	75,9	7,6	11,4	5,1
	Merkel cell carcinoma	48	1	0,0	0,0	100,0	0,0
Tumors of the head and neck	Squamous cell carcinoma of the larynx	109	99	99,0	0,0	1,0	0,0
	Squamous cell carcinoma of the pharynx	60	57	98,2	1,8	0,0	0,0
	Oral squamous cell carcinoma (floor of the mouth)	130	121	96,7	0,0	3,3	0,0
	Pleomorphic adenoma of the parotid gland	50	45	100,0	0,0	0,0	0,0
	Warthin tumor of the parotid gland	104	46	100,0	0,0	0,0	0,0
	Basal cell adenoma of the salivary gland	101	15	100,0	0,0	0,0	0,0
Tumors of the lung, pleura and thymus	Adenocarcinoma of the lung	246	179	100,0	0,0	0,0	0,0
	Squamous cell carcinoma of the lung	130	73	97,3	2,7	0,0	0,0
	Mesothelioma, epithelioid	40	30	83,3	3,3	6,7	6,7
	Mesothelioma, biphasic	77	24	79,2	0,0	12,5	8,3
	Thymoma	29	27	100,0	0,0	0,0	0,0
	Lung, neuroendocrine tumor (NET)	29	29	72,4	6,9	17,2	3,4
Tumors of the female genital tract	Squamous cell carcinoma of the vagina	78	29	93,1	3,4	3,4	0,0
	Squamous cell carcinoma of the vulva	157	99	97,0	2,0	1,0	0,0
	Squamous cell carcinoma of the cervix	315	87	95,4	2,3	2,3	0,0
	Adenocarcinoma of the cervix	56	22	90,9	4,5	4,5	0,0
	Endometrioid endometrial carcinoma	338	255	96,1	0,0	3,1	0,8
	Endometrial serous carcinoma	86	30	50,0	0,0	36,7	13,3
	Carcinosarcoma of the uterus	57	55	72,7	5,5	9,1	12,7
	Endometrial carcinoma, high grade, G3	13	13	92,3	7,7	0,0	0,0
	Endometrial clear cell carcinoma	9	8	50,0	0,0	25,0	25,0
	Endometrioid carcinoma of the ovary	130	78	79,5	7,7	11,5	1,3
	Serous carcinoma of the ovary	580	459	66,9	9,2	19,0	5,0
	Mucinous carcinoma of the ovary	101	57	100,0	0,0	0,0	0,0
	Clear cell carcinoma of the ovary	51	47	85,1	8,5	4,3	2,1
	Carcinosarcoma of the ovary	47	40	90,0	5,0	5,0	0,0
	Granulosa cell tumor of the ovary	44	41	100,0	0,0	0,0	0,0
	Leydig cell tumor of the ovary	4	4	100,0	0,0	0,0	0,0
	Sertoli cell tumor of the ovary	1	1	100,0	0,0	0,0	0,0
	Sertoli Leydig cell tumor of the ovary	3	3	100,0	0,0	0,0	0,0
	Steroid cell tumor of the ovary	3	3	100,0	0,0	0,0	0,0
	Brenner tumor	41	28	100,0	0,0	0,0	0,0
Tumors of the breast	Invasive breast carcinoma of no special type	1810	475	97,3	1,3	0,8	0,6
	Lobular carcinoma of the breast	364	132	99,2	0,0	0,8	0,0
	Medullary carcinoma of the breast	34	8	75,0	0,0	12,5	12,5
	Tubular carcinoma of the breast	29	2	100,0	0,0	0,0	0,0
	Mucinous carcinoma of the breast	65	6	100,0	0,0	0,0	0,0
Tumors of the digestive system	Adenomatous polyp, low-grade dysplasia	50	20	100,0	0,0	0,0	0,0
	Adenomatous polyp, high-grade dysplasia	50	34	100,0	0,0	0,0	0,0
	Adenocarcinoma of the colon	2533	2177	87,0	2,3	8,7	1,9
	Gastric adenocarcinoma, diffuse type	265	177	99,4	0,0	0,6	0,0
	Gastric adenocarcinoma, intestinal type	265	176	89,2	1,7	9,1	0,0
	Gastric adenocarcinoma, mixed type	62	48	93,8	0,0	4,2	2,1
	Adenocarcinoma of the esophagus	133	78	88,5	3,8	5,1	2,6
	Squamous cell carcinoma of the esophagus	125	70	95,7	0,0	2,9	1,4
	Squamous cell carcinoma of the anal canal	91	67	100,0	0,0	0,0	0,0
	Cholangiocarcinoma	121	38	97,4	0,0	2,6	0,0
	Gallbladder adenocarcinoma	51	42	92,9	0,0	4,8	2,4
	Gallbladder Klatskin tumor	42	29	100,0	0,0	0,0	0,0
	Hepatocellular carcinoma	312	259	97,3	1,2	1,2	0,4
	Ductal adenocarcinoma of the pancreas	709	430	95,1	2,1	1,9	0,9
	Pancreatic/Ampullary adenocarcinoma	128	64	85,9	3,1	10,9	0,0
	Acinar cell carcinoma of the pancreas	18	17	100,0	0,0	0,0	0,0
	Gastrointestinal stromal tumor (GIST)	62	55	100,0	0,0	0,0	0,0
	Appendix, neuroendocrine tumor (NET)	25	16	81,3	12,5	6,3	0,0
	Colorectal, neuroendocrine tumor (NET)	12	9	100,0	0,0	0,0	0,0
	Ileum, neuroendocrine tumor (NET)	53	49	100,0	0,0	0,0	0,0
	Pancreas, neuroendocrine tumor (NET)	101	80	92,5	1,3	6,3	0,0
	Colorectal, neuroendocrine carcinoma (NEC)	14	11	72,7	18,2	9,1	0,0
	Ileum, neuroendocrine carcinoma (NEC)	8	7	100,0	0,0	0,0	0,0
	Gallbladder, neuroendocrine carcinoma (NEC)	4	4	50,0	0,0	50,0	0,0
	Pancreas, neuroendocrine carcinoma (NEC)	14	14	78,6	0,0	21,4	0,0
Tumors of the urinary system	Non-invasive papillary urothelial carcinoma, pTa G2 low grade	264	78	100,0	0,0	0,0	0,0
	Non-invasive papillary urothelial carcinoma, pTa G2 high grade	221	71	98,6	0,0	1,4	0,0
	Non-invasive papillary urothelial carcinoma, pTa G3	313	112	100,0	0,0	0,0	0,0
	Urothelial carcinoma, pT2-4 G3	1318	514	89,7	2,5	6,2	1,6
	Squamous cell carcinoma of the bladder	22	20	100,0	0,0	0,0	0,0
	Small cell neuroendocrine carcinoma of the bladder	23	5	60,0	0,0	40,0	0,0
	Sarcomatoid urothelial carcinoma	25	22	90,9	0,0	0,0	9,1
	Urothelial carcinoma of the kidney pelvis	62	54	96,3	0,0	3,7	0,0
	Clear cell renal cell carcinoma	1655	1086	91,8	6,7	0,9	0,6
	Papillary renal cell carcinoma	433	290	91,7	0,3	5,2	2,8
	Clear cell (tubulo) papillary renal cell carcinoma	33	18	88,9	5,6	5,6	0,0
	Chromophobe renal cell carcinoma	190	139	92,1	1,4	0,7	5,8
	Oncocytoma of the kidney	279	209	93,8	0,0	2,9	3,3
Tumors of the male genital organs	Adenocarcinoma of the prostate, Gleason 3+3	83	82	100,0	0,0	0,0	0,0
	Adenocarcinoma of the prostate, Gleason 4+4	80	78	100,0	0,0	0,0	0,0
	Adenocarcinoma of the prostate, Gleason 5+5	85	85	100,0	0,0	0,0	0,0
	Adenocarcinoma of the prostate (recurrence)	258	227	100,0	0,0	0,0	0,0
	Small cell neuroendocrine carcinoma of the prostate	19	2	50,0	0,0	0,0	50,0
	Seminoma	682	625	98,2	1,6	0,2	0,0
	Embryonal carcinoma of the testis	54	39	100,0	0,0	0,0	0,0
	Leydig cell tumor of the testis	31	29	100,0	0,0	0,0	0,0
	Sertoli cell tumor of the testis	2	1	100,0	0,0	0,0	0,0
	Sex cord stromal tumor of the testis	1	1	100,0	0,0	0,0	0,0
	Spermatocytic tumor of the testis	1	1	100,0	0,0	0,0	0,0
	Yolk sac tumor	53	37	94,6	0,0	5,4	0,0
	Teratoma	53	24	91,7	0,0	8,3	0,0
	Squamous cell carcinoma of the penis	92	86	100,0	0,0	0,0	0,0
Tumors of endocrine organs	Adenoma of the thyroid gland	113	63	100,0	0,0	0,0	0,0
	Papillary thyroid carcinoma	391	329	99,7	0,0	0,3	0,0
	Follicular thyroid carcinoma	154	105	100,0	0,0	0,0	0,0
	Medullary thyroid carcinoma	111	55	98,2	1,8	0,0	0,0
	Parathyroid gland adenoma	43	39	100,0	0,0	0,0	0,0
	Anaplastic thyroid carcinoma	45	19	94,7	0,0	5,3	0,0
	Adrenal cortical adenoma	48	41	100,0	0,0	0,0	0,0
	Adrenal cortical carcinoma	27	17	100,0	0,0	0,0	0,0
	Pheochromocytoma	51	44	4,5	4,5	29,5	61,4
Tumors of hematopoetic and lymphoid tissues	Hodgkin’s lymphoma	103	98	99,0	0,0	1,0	0,0
	Small lymphocytic lymphoma, B-cell type (B-SLL/B-CLL)	50	49	100,0	0,0	0,0	0,0
	Diffuse large B cell lymphoma (DLBCL)	113	112	91,1	2,7	5,4	0,9
	Follicular lymphoma	88	87	97,7	0,0	2,3	0,0
	T-cell non-Hodgkin’s lymphoma	25	25	100,0	0,0	0,0	0,0
	Mantle cell lymphoma	18	18	100,0	0,0	0,0	0,0
	Marginal zone lymphoma	16	16	100,0	0,0	0,0	0,0
	Diffuse large B-cell lymphoma (DLBCL) in the testis	16	16	100,0	0,0	0,0	0,0
	Burkitt lymphoma	5	5	100,0	0,0	0,0	0,0
	Granular cell tumor	53	17	5,9	0,0	0,0	94,1
	Leiomyoma	50	48	100,0	0,0	0,0	0,0
	Leiomyosarcoma	94	90	100,0	0,0	0,0	0,0
	Liposarcoma	145	93	100,0	0,0	0,0	0,0
	Malignant peripheral nerve sheath tumor (MPNST)	15	15	86,7	6,7	6,7	0,0
	Myofibrosarcoma	26	26	100,0	0,0	0,0	0,0
	Angiosarcoma	74	36	97,2	0,0	2,8	0,0
	Angiomyolipoma	91	87	100,0	0,0	0,0	0,0
	Dermatofibrosarcoma protuberans	21	18	100,0	0,0	0,0	0,0
	Ganglioneuroma	14	14	7,1	0,0	21,4	71,4
	Kaposi sarcoma	8	5	100,0	0,0	0,0	0,0
	Neurofibroma	117	106	59,4	25,5	12,3	2,8
	Sarcoma, not otherwise specified (NOS)	74	70	95,7	0,0	4,3	0,0
	Paraganglioma	41	39	0,0	7,7	25,6	66,7
	Ewing sarcoma	23	15	100,0	0,0	0,0	0,0
	Rhabdomyosarcoma	7	7	85,7	0,0	0,0	14,3
	Schwannoma	122	116	11,2	5,2	19,8	63,8
	Synovial sarcoma	12	11	100,0	0,0	0,0	0,0
	Osteosarcoma	44	17	100,0	0,0	0,0	0,0
	Chondrosarcoma	40	12	100,0	0,0	0,0	0,0
	Rhabdoid tumor	5	5	80,0	0,0	20,0	0,0
	Solitary fibrous tumor	17	17	100,0	0,0	0,0	0,0

L1CAM: L1 cell adhesion molecule; TMA: tissue microarray.

**Table 2:** L1CAM immunostaining and tumor phenotype.

		n	L1CAM immunostaining				P
			negative (%)	weak (%)	moderate (%)	strong (%)	
Clear cell renal cell carcinoma	ISUP 1	243	85,6	3,7	10,3	0,4	0,0014
	ISUP 2	364	93,1	1,4	5,5	0,0	
	ISUP 3	239	93,7	1,7	3,3	1,3	
	ISUP 4	74	90,5	1,4	4,1	4,1	
	Fuhrman 1	61	77,0	6,6	14,8	1,6	<0.0001
	Fuhrman 2	616	92,0	1,9	6,0	0,0	
	Fuhrman 3	266	93,6	1,1	3,4	1,9	
	Fuhrman 4	87	90,8	2,3	3,4	3,4	
	Thoenes 1	324	88,9	3,4	7,4	0,3	0,0431
	Thoenes 2	437	92,7	1,6	5,0	0,7	
	Thoenes 3	97	89,7	2,1	4,1	4,1	
	UICC 1	310	88,4	4,2	7,1	0,3	0,0976
	UICC 2	35	94,3	0,0	2,9	2,9	
	UICC 3	87	87,4	1,1	9,2	2,3	
	UICC 4	69	92,8	0,0	5,8	1,4	
	pT1	611	90,8	2,9	5,9	0,3	0,0364
	pT2	120	95,0	1,7	2,5	0,8	
	pT3-4	306	91,5	0,7	6,2	1,6	
	pN0	152	90,1	0,7	8,6	0,7	0,6900
	pN+	26	96,2	0,0	3,8	0,0	
	pM0	100	86,0	2,0	10,0	2,0	0,4592
	pM+	87	90,8	1,1	4,6	3,4	


Papillary renal cell carcinoma	ISUP 1	37	97,3	0,0	2,7	0,0	0,4662
	ISUP 2	119	92,4	1,7	0,8	5,0	
	ISUP 3	66	89,4	3,0	4,5	3,0	
	ISUP 4	5	100,0	0,0	0,0	0,0	
	Fuhrman 1	3	100,0	0,0	0,0	0,0	0,8732
	Fuhrman 2	164	93,3	0,6	2,4	3,7	
	Fuhrman 3	68	91,2	2,9	2,9	2,9	
	Fuhrman 4	9	88,9	0,0	11,1	0,0	
	Thoenes 1	54	98,1	0,0	1,9	0,0	0,0841
	Thoenes 2	137	92,7	1,5	1,5	4,4	
	Thoenes 3	14	85,7	0,0	14,3	0,0	
	UICC 1	91	95,6	0,0	2,2	2,2	0,1880
	UICC 2	16	93,8	0,0	6,3	0,0	
	UICC 3	4	100,0	0,0	0,0	0,0	
	UICC 4	9	66,7	0,0	11,1	22,2	
	pT1	179	93,9	2,8	1,7	1,7	0,0940
	pT2	44	90,9	0,0	4,5	4,5	
	pT3-4	27	77,8	3,7	7,4	11,1	
	pN0	18	88,9	0,0	5,6	5,6	0,1721
	pN+	13	69,2	7,7	23,1	0,0	
	pM0	23	95,7	0,0	4,3	0,0	0,0464
	pM+	11	63,6	9,1	9,1	18,2	


Urothelial bladder carcinoma	pTa G2 low	353	100,0	0,0	0,0	0,0	0,0640
	pTa G2 high	153	98,7	1,3	0,0	0,0	
	pTa G3	96	100,0	0,0	0,0	0,0	
	pT2	376	90,2	6,4	0,8	2,7	0.9062*
	pT3	484	88,6	7,0	1,2	3,1	
	pT4	241	88,4	6,6	2,1	2,9	
	G2	87	95,4	2,3	0,0	2,3	0.9210*
	G3	992	88,6	7,1	1,4	2,9	
	pN0	521	87,3	8,4	1,3	2,9	0.1488*
	pN+	359	91,4	4,7	0,8	3,1	


Adenocarcinoma of the pancreas	pT1	15	53,3	33,3	13,3	0,0	0,1027
	pT2	72	41,7	29,2	15,3	13,9	
	pT3	321	61,1	23,4	10,6	5,0	
	pT4	34	52,9	29,4	11,8	5,9	
	G1	16	43,8	25,0	18,8	12,5	0,5170
	G2	312	55,1	26,0	12,5	6,4	
	G3	93	64,5	21,5	7,5	6,5	
	pN0	96	59,4	21,9	12,5	6,3	0,8568
	pN+	345	56,2	26,1	11,3	6,4	
	MMR proficient	388	56,4	25,5	11,9	6,2	0,3321
	MMR deficient	3	100,0	0,0	0,0	0,0	
Adenocarcinoma of the stomach	pT1-2	42	88,1	9,5	2,4	0,0	0,1991
	pT3	101	82,2	11,9	4,0	2,0	
	pT4	95	93,7	4,2	2,1	0,0	
	pN0	60	91,7	8,3	0,0	0,0	0,1437
	pN+	178	86,0	9,0	3,9	1,1	
	MMR proficient	207	85,5	10,1	2,9	1,4	0,9461
	MMR deficient	29	96,6	0,0	3,4	0,0	


Endometrioid endometrial carcinoma	pT1	105	95,2	4,8	0,0	0,0	0,3796
	pT2	24	95,8	4,2	0,0	0,0	
	pT3-4	36	91,7	2,8	2,8	2,8	
	pN0	49	95,9	2,0	0,0	2,0	0,3879
	pN+	30	93,3	3,3	3,3	0,0	


Serous carcinoma of the ovary	pT1	29	75,9	17,2	0,0	6,9	0,1105
	pT2	41	58,5	29,3	2,4	9,8	
	pT3	246	54,5	32,5	7,3	5,7	
	pN0	81	65,4	23,5	6,2	4,9	0,3687
	pN1	156	53,8	33,3	7,1	5,8	


Adenocarcinoma of the colon	pT1	77	81,8	13,0	5,2	0,0	0,1943
	pT2	410	78,5	16,6	2,0	2,9	
	pT3	1162	81,0	14,6	2,6	1,8	
	pT4	419	84,5	11,7	1,9	1,9	
	pN0	1076	80,8	15,1	2,3	1,9	0,6921
	pN+	985	82,1	13,3	2,4	2,1	
	V0	1503	81,2	14,4	2,3	2,1	0,9517
	V1	529	81,7	13,8	2,6	1,9	
	L0	684	81,9	14,5	2,2	1,5	0,6334
	L1	1357	81,4	14,0	2,3	2,3	
	right side	442	85,1	12,0	1,1	1,8	0,0104
	left side	1175	78,7	15,9	3,1	2,2	
	MMR proficient	1127	78,5	16,6	2,8	2,0	0,0064
	MMR deficient	83	92,8	4,8	1,2	1,2	
	RAS wildtype	429	80,0	15,9	2,6	1,6	0,5177
	RAS mutation	340	78,2	16,5	3,2	2,1	
	BRAF wildtype	128	82,0	14,1	2,3	1,6	0,5779
	BRAF V600E mutation	21	90,5	9,5	0,0	0,0	

* only in pT2-4 urothelial bladder carcinomas, abbreviation: pT: pathological tumor stage, G: Grade, pN: pathological lymph node status, pM: pathological status of distant metastasis, MMR: mismatch repair, ISUP: International Socity of Urological Pathology, UICC: Union for Internatinal Cancer Control, V: venous invasion, L: lymphatic invasion, BRAF: B-Raf Proto-Oncogene, RAS: RAS Oncogene family

## Discussion and conclusion

**Figure 4:**
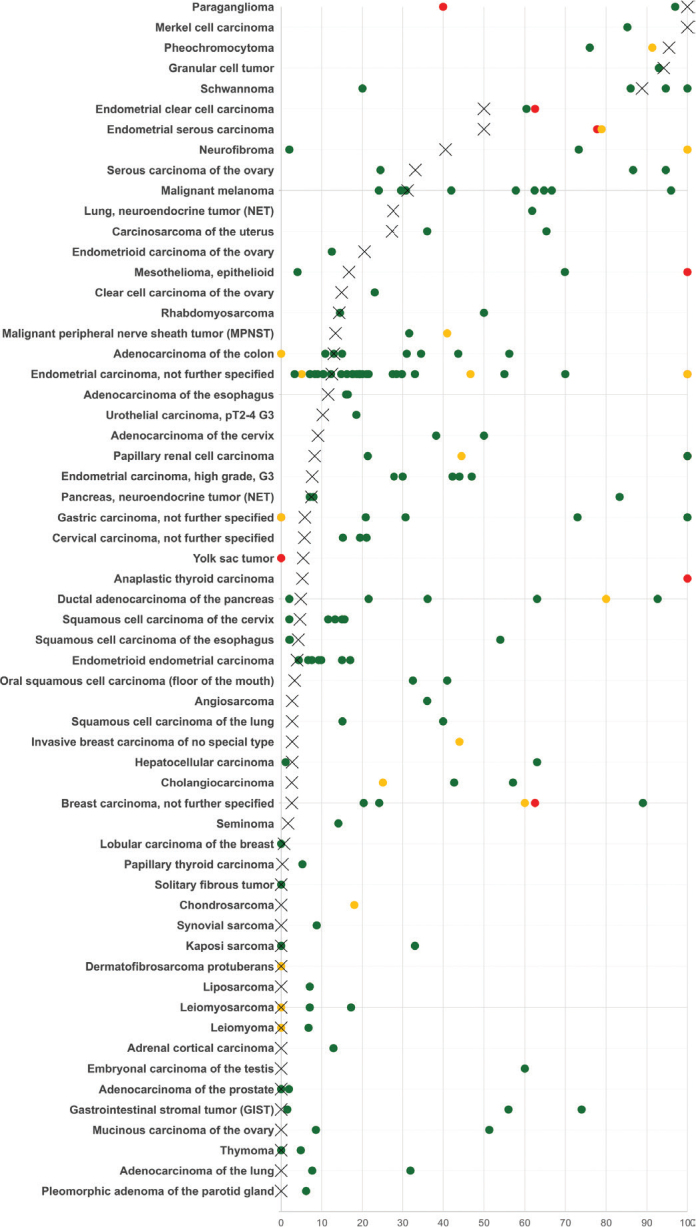
Comparison of L1CAM immunostaining results with previous L1CAM studies. An ‘X’ indicates the fraction of L1CAM positive cancers in the present study, dots indicate the reported frequencies from the literature for comparison: red dots mark studies with ≤ 10 tumors analyzed, yellow dots mark studies with ≥ 11 ≤ 25 tumors analyzed and green dots mark studies with > 25 tumors analyzed. All studies are listed in Supplementary Table 1. L1CAM: L1 cell adhesion molecule.

Our analysis of 12,888 tumors from 135 different tumor categories provides a comprehensive overview of L1CAM expression on tumor cells in different cancer types. Considering the heterogeneity of literature data (summarized in Figure 4), such information could hardly be compiled from published studies. That particularly high L1CAM positivity rates were observed on tumor cells in neural and neuroendocrine cancers fits with the expression of L1CAM in corresponding normal tissues and with data from the literature [5, 6]. Considering the absence of L1CAM expression in corresponding normal cell types, L1CAM tumor cell positivity of most other tumor types might reflect neo-expression of the protein. Despite conflicting literature data, particularly high rates of L1CAM tumor cell positivity in tumors arising from endometrium, ovary, endocervix, mesothelium, the gastrointestinal and the biliopancreatic tract, as well as in malignant melanoma are consistent with at least a fraction of previous studies (Table 3). The ranking order of tumor entities according to their rate of L1CAM tumor cell expression and the observation that high-level L1CAM expression on tumor cells can occur in more than 40 different tumor categories, represent the key results of this study. L1CAM belongs to a group of potential new molecules in the field of targeted cancer therapy, and several approaches targeting L1CAM are currently being evaluated, including neutralizing antibodies [23], bispecific antibodies [24], radioimmunoconjugates [25], and CAR-T cells [26]. Our ranking of L1CAM expression prevalence highlights the most promising tumor entities that could potentially benefit from new therapies targeting L1CAM.

The availability of several large cohorts of tumors of the same histological type enabled us to analyze the relationship between L1CAM expression on tumor cells and parameters of cancer aggressiveness in this study. However, the significantly higher rate of L1CAM positivity in muscle-invasive than in non-invasive urothelial carcinoma was the only association found between L1CAM tumor cell expression and aggressive tumor phenotype. The absence of significant associations with histological parameters of dismal prognosis in clear cell and papillary RCC, pT2–4 urothelial carcinoma of the bladder, adenocarcinomas of the pancreas, the colorectum and the stomach, endometrioid endometrial carcinoma and serous high-grade carcinoma of the ovary argues against a major cancer driving role of L1CAM. In correlation with our data, others have also failed to find associations with aggressive tumor phenotype or poor prognosis in cohorts of endometrial carcinoma [29], squamous cell carcinoma of the cervix [30] and the oral cavity [31], ductal adenocarcinoma of the pancreas [32], clear cell carcinoma of the ovary [33], adenocarcinoma and squamous cell carcinoma of the esophagus [34], GIST [35] and neuroblastoma [22].

However, it is of note that a significant number of other studies have highlighted a role of L1CAM in cancer progression and described associations between high L1CAM expression and unfavorable prognostic tumor features [36]. For example, L1CAM expression on tumor cells was linked to aggressive cancer features and poor patient outcome in endometrial carcinoma [13, 37–40], oral squamous cell carcinoma [14], uterine mesonephric-like adenocarcinoma [41], breast cancer [42], endometrioid carcinoma of the ovary [43], ductal adenocarcinoma of the pancreas [19], colorectal cancer [44], cholangiocarcinoma [45], hepatocellular carcinoma [46], carcinosarcoma of the uterus [15], serous carcinoma of the ovary [17], adenocarcinoma of the lung [18] and esophageal squamous cell carcinoma [20]. Several, functional studies have linked high L1CAM expression to increased tumor growth in colorectal cancer cells [47], increased cell proliferation, cell migration, and cell invasion in squamous carcinoma [14] and gastric cancer cells [7], higher motility and proliferation in glioblastoma cells [48], and enhanced transendothelial migration (TEM) in a pancreatic cancer model [49]. Enforced downregulation of L1CAM was associated with reduced cell migration in melanoma cell lines [50], inhibition of cell proliferation, cell invasion, and induction of cell cycle arrest in pancreatic cancer cell lines [51] and decreased tumor growth as well as increased survival of glioma-bearing animals.

In view of these conflicting data, we are confident in our IHC data because our assay was extensively validated according to the recommendations of the International Working Group of Antibody Validation (IWGAV) [52] by comparing our IHC findings in normal tissues with data obtained using another independent anti-L1CAM antibody, as well as with published normal tissue RNA data from three different databases [53–56]. To test a broad range of proteins and their relevant posttranslational modifications for a possible cross-reactivity, 76 different normal tissue categories were included in this analysis. The validity of our IHC assay was supported by the detection of L1CAM positivity by IHC in all tissues with documented L1CAM RNA expression, including adrenal gland, pituitary gland, prostate, kidney, seminal vesicles, and the gastrointestinal tract. While L1CAM RNA expression was not detected in the fallopian tube, for which a distinct staining of a subset of cells was observed by our assay, fallopian tube staining was confirmed by the independent L1CAM antibody 14.10, which also confirmed all other normal cell staining of our assay. The critical impact of assays for IHC studies has also been illustrated in an earlier analysis of 5,833 tumors from 128 tumor entities where a low sensitivity assay using the L1CAM antibody UJ127 resulted in positive cases in only 28 of 128 tumor entities (our study: 74 of 135) and a L1CAM positivity in only 5% of 22 endometrial carcinomas (our study: 12.5%). It is of note that our IHC assay was designed to detect L1CAM protein on tumor cells. Cleaved or exosomal fragments of L1CAM protein, which are formed under certain conditions [57], cannot be detected by IHC and thus did not influence our data.

In summary, our data show that L1CAM can be expressed in a broad range of different tumor entities, while its expression level is largely unrelated to cancer aggressiveness. Our ranking list of tumor entities according to their rate of L1CAM positivity helps to define groups of cancer patients which could best benefit from anti-L1CAM therapies.

## Supplementary Material



## Data Availability

All data generated or analyzed during this study are included in this published article.
