# Supplementary material for: L1CAM/CD171 expression in human tumors and its association with tumor phenotype
Source: Acta Oncol. 2025 Dec 2;64:43587. doi: 10.2340/1651-226X.2025.43587 (PMC12681027; doi:10.2340/1651-226X.2025.43587)
Supplement: Supplementary file 1 [file AO-64-43587-s1.pdf]

Supplementary material has been published as submitted. It has not been copyedited, or typeset by Acta Oncologica

Supplementary Figure 1: **Assay validation by comparison of two antibodies. The panel shows immunostaining results obtained by two independent L1CAM antibodies.** Using MSVA-171R, immunostaining was seen in the nerve fibers of appendix muscular wall (A), subsets of B-cells and monocytic cells of tonsil (B), a subset of collecting duct cells of kidney medulla (C), a small subset of the epithelial cells of the fallopian tube (D), the posterior lobe of the pituitary gland (E), and the grey matter of the cerebrum (F), whereas no staining was seen in the submandibular gland (except nerve fibers) (G), and the liver (H). Using clone 14.10, a comparable staining was seen in the appendix muscular wall (a), tonsil (b), kidney (c), fallopian tube (d), pituitary gland (e), cerebrum (f), submandibular gland (g) and the liver (h). The images A-H and a-h are from consecutive tissue sections.

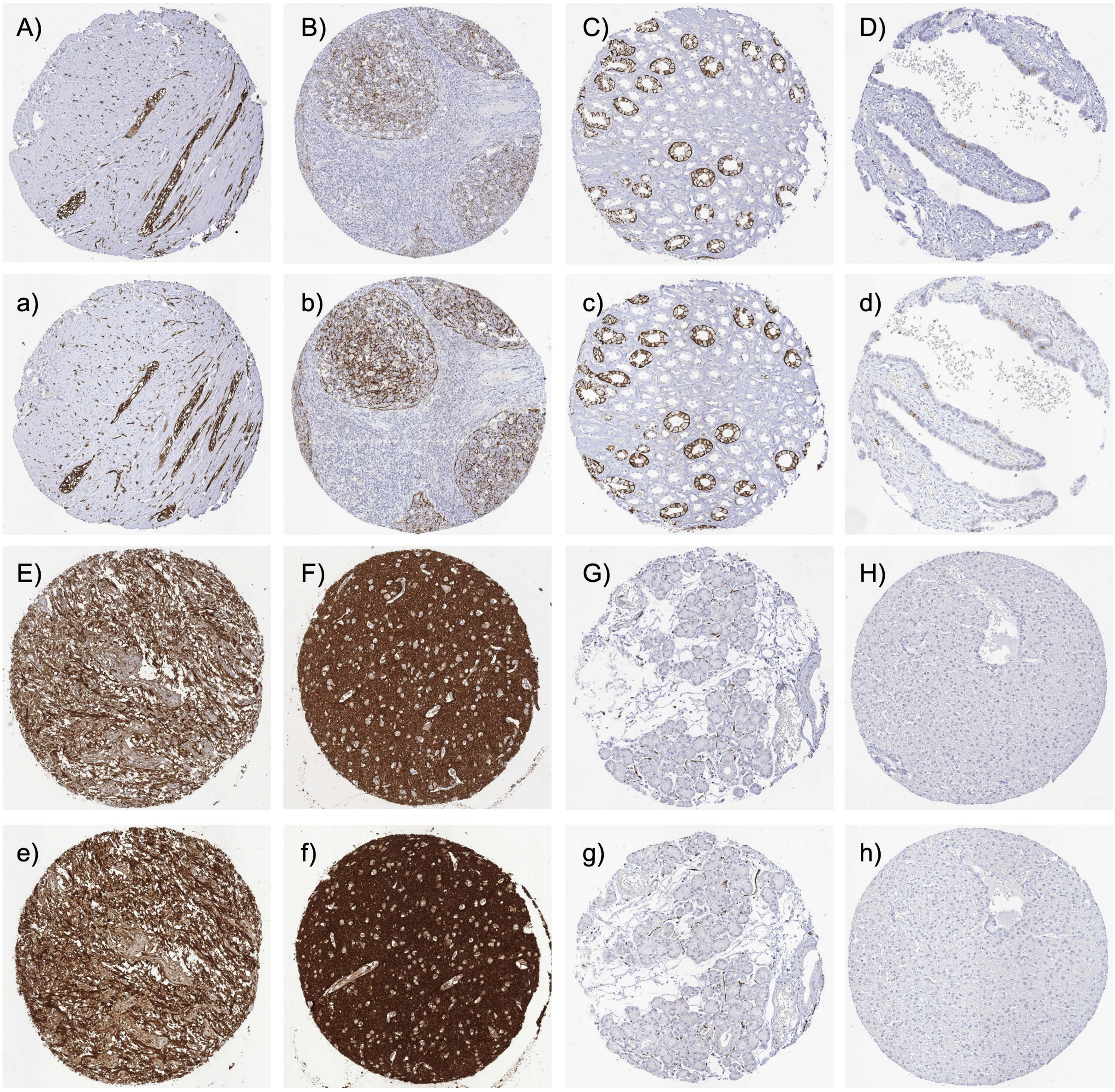
A)
B)
C)
D)
a)
b)
c)
d)
E)
F)
G)
H)
e)
f)
g)
h)

Supplementary Table 3: L1CAM immunostaining in previous studies

Author & Year	Pubmed ID	Tumor entity	Number of tumors	Positive
Vermij 2023	36690721	Endometrial carcinoma, high grade, G3	648	27.80%
Horeweg 2022	35296668	Endometrial cancer	378	27.40%
Joe 2023	37835593	Endometrial cancer	183	10.40%
Kim 2023	37236032	Endometrioid endometrial carcinoma	140	9.90%
Asano 2020	31792716	Endometrial cancer	161	29.80%
Corrado 2018	29980240	Endometrial cancer	113	70.00%
Romanova 2023	37652517	Squamous cell carcinoma of the cervix	154	15.60%
Kim 2023	36825596	Oral squamous cell carcinoma (floor of the mouth)	80	32.50%
Mamale 2023	37116166	Endometrial cancer	49	28.50%
Lopes da Silva 2023	37200263	Carcinosarcoma of the uterus	57	36.10%
Zeller 2021	34659530	Endometrial cancer	212	19.30%
Paulo Mancusi de Carvalho 2020	32366597	Adenocarcinoma of the cervix	26	50.00%
Paulo Mancusi de Carvalho 2020	32366597	Squamous cell carcinoma of the cervix	45	15.00%
Van Gool 2016	26743472	Endometrial carcinoma, high grade, G3	116	44.00%
Smogeli 2016	27488577	Endometrial cancer	388	9.00%
Schirmer 2013	23530769	Endometrial cancer	5	100.00%
Yoon 2024	38309030	Endometrial cancer	335	10.40%
Vinklerova 2021	34356280	Endometrial cancer	249	7.20%
Vinklerova 2022	34356281	Squamous cell carcinoma of the cervix	249	11.60%
Bergmann 2010	20811670	Ductal adenocarcinoma of the pancreas	110	92.70%
Notaro 2016	27233077	Endometrial cancer	50	20.00%
Versluis 2018	30140948	Carcinosarcoma of the uterus	81	65.40%
Liszka 2021	35048633	Pancreas, neuroendocrine tumor (NET)	29	7.00%
Romani 2022	35429348	Endometrial carcinoma, high grade, G3	55	47.00%
Visser 2021	34418427	Endometrioid endometrial carcinoma	302	4.30%
Zeimet 2013	23781004	Endometrial cancer	1021	17.70%
Dos Santos 2023	37478615	Serous carcinoma of the ovary	644	94.70%
Chopra 2024	39067486	Adenocarcinoma of the cervix	50	38.30%
Chopra 2025	39067486	Squamous cell carcinoma of the cervix	214	13.30%
Adnan Ali 2022	36066622	Ductal adenocarcinoma of the pancreas	62	63.00%
Reijnen 2020	32413043	Endometrial cancer	763	10.40%
Yu 2019	31934096	Adenocarcinoma of the lung	72	31.94%
Yu 2020	31934097	Squamous cell carcinoma of the lung	50	40.00%
Maccio 2022	36142656	Malignant melanoma	47	96.00%
Loukovaara 2024	38972163	Endometrial cancer	748	14.80%
Geels 2016	26891628	Endometrioid endometrial carcinoma	92	7.60%
Soovares 2022	34711431	Endometrioid carcinoma of the ovary	201	12.43%
Müerköster 2007	17086212	Ductal adenocarcinoma of the pancreas	20	80.00%
Kim 2023	37797754	Papillary renal cell carcinoma	43	100.00%
Tsutsumi 2011	21360711	Ductal adenocarcinoma of the pancreas	107	21.50%
Klat 2019	30591489	Endometrial cancer	312	29.80%
Bosse 2014	25126672	Endometrial cancer	865	7.00%
Suh 2014	25337993	Endometrial cancer	68	19.10%
Adnan 2022	35296132	Oral squamous cell carcinoma (floor of the mouth)	100	41.00%
Soovares 2017	28625395	Endometrioid endometrial carcinoma	249	15.00%
Soovares 2017	28625395	Clear cell carcinoma of the ovary	140	23.00%
Nova-Camacho 2023	36170616	Papillary renal cell carcinoma	8	100.00%
de Freitas 2018	30557309	Endometrioid endometrial carcinoma	47	17.00%
Kommoss 2018	30050154	Endometrial cancer	452	21.50%
Ravagi 2022	36358847	Endometrial carcinoma, high grade, G3	94	30.00%
Yamazaki 2022	35707843	Endometrial cancer	123	18.60%
Yamazaki 2022	35707843	Endometrial carcinoma, high grade, G3	26	42.30%
Yamazaki 2022	35707843	Endometrial seous carcinoma	9	77.77%
Yamazaki 2022	35707843	Endometrial clear cell carcinoma	8	62.50%
Němejcová 2023	36855066	Serous carcinoma of the ovary	250	24.40%
Kim 2012	22472175	Anaplastic thyroid carcinoma	9	100.00%
Kim 2020	32331700	Endometrial clear cell carcinoma	48	60.40%
Pasanen 2020	32060377	Endometrioid endometrial carcinoma	657	9.28%
Suarez-Arroyo 2016	27648361	Invasive breast carcinoma of no special type	24	44.00%
Vrede 2021	33858677	Endometrial cancer	763	10.40%
Huvila 2018	29486992	Endometrioid endometrial carcinoma	305	6.60%
Al-Obaidy 2019	31135486	Papillary renal cell carcinoma	18	44.44%
Ferreira 2024	38430556	Endometrial cancer	236	3.38%
Yamada 2022	34988657	Thymoma	94	0.00%
Rawnaq 2009	19414364	Adenocarcinoma of the esophagus	116	16.37%
Rawnaq 2009	19414364	Squamous cell carcinoma of the esophagus	141	2.12%
Rawnaq 2012	21195422	Squamous cell carcinoma of the cervix	44	2.00%
Rawnaq 2012	21195422	Chondrosarcoma	11	18.00%
Rawnaq 2012	21195422	Adenocarcinoma of the esophagus	116	16.00%
Rawnaq 2012	21195422	Squamous cell carcinoma of the esophagus	141	2.00%
Rawnaq 2012	21195422	Gastrointestinal stromal tumor (GIST)	103	56.00%
Rawnaq 2012	21195422	Granular cell tumor	28	93.00%
Rawnaq 2012	21195422	Kaposi sarcoma	30	33.00%
Rawnaq 2012	21195422	Leiomyosarcoma	129	7.00%
Rawnaq 2012	21195422	Liposarcoma	104	7.00%
Rawnaq 2012	21195422	Malignant melanoma	50	24.00%
Rawnaq 2012	21195422	Schwannoma	54	20.00%
Rawnaq 2012	21195422	Mesothelioma, epithelioid	28	4.00%
Rawnaq 2012	21195422	Neurofibroma	43	2.00%
Rawnaq 2012	21195422	Ductal adenocarcinoma of the pancreas	111	2.00%
Rawnaq 2012	21195422	Pancreas, neuroendocrine tumor (NET)	63	8.00%
Min 2010	20501614	Cholangiosarcoma	42	57.14%
Guo 2012	22888955	Hepatocellular carcinoma	130	63.08%
Gavert 2005	15716380	Adenocarcinoma of the colon	25	0.00%
Ben 2010	20162456	Ductal adenocarcinoma of the pancreas	94	36.20%
Li 2009	19920102	Cholangiosarcoma	75	42.70%
Lee 2010	19865098	Malignant melanoma	173	62.00%
Wiedenhoefer 2023	36852510	Mucinous carcinoma of the ovary	39	51.30%
Inaguma 2016	27419370	Schwannoma	74	100.00%
Inaguma 2016	27419370	Paraganglioma	67	97.00%
Inaguma 2016	27419370	Merkel cell carcinoma	27	85.20%
Inaguma 2016	27419370	Pancreas, neuroendocrine tumor (NET)	42	83.30%
Inaguma 2016	27419370	Neurofibroma	38	73.30%
Inaguma 2016	27419370	Malignant melanoma	88	64.80%
Inaguma 2016	27419370	Malignant peripheral nerve sheath tumor (MPNST)	57	31.60%
Inaguma 2016	27419370	Malignant melanoma	65	30.80%
Inaguma 2016	27419370	Malignant melanoma	27	29.60%
Inaguma 2016	27419370	Schwannoma	38	94.70%
Inaguma 2016	27419370	Malignant melanoma	42	66.70%
Inaguma 2016	27419370	Malignant melanoma	64	57.80%
Inaguma 2016	27419370	Rhabdomyosarcoma	42	50.00%
Inaguma 2016	27419370	Angiosarcoma	97	36.10%
Inaguma 2016	27419370	Rhabdomyosarcoma	55	14.50%
Inaguma 2016	27419370	Synovial sarcoma	34	8.80%
Inaguma 2016	27419370	Kaposi sarcoma	35	0.00%
Inaguma 2016	27419370	Solitary fibrous tumor	28	0.00%
Inaguma 2016	27419370	Gastrointestinal stromal tumor (GIST)	1300	1.50%
Inaguma 2016	27419370	Leiomyosarcoma	29	17.20%
Inaguma 2016	27419370	Liposarcoma	45	6.70%
Inaguma 2016	27419370	Serous carcinoma of the ovary	97	86.60%
Inaguma 2016	27419370	Mesothelioma, epithelioid	183	69.90%
Inaguma 2016	27419370	Embryonal carcinoma of the testis	35	60.00%
Inaguma 2016	27419370	Adenocarcinoma of the colon	210	56.20%
Inaguma 2016	27419370	Endometrial cancer	97	33.00%
Inaguma 2016	27419370	Cholangiosarcoma	20	25.00%
Inaguma 2016	27419370	Papillary renal cell carcinoma	333	21.30%
Inaguma 2016	27419370	Urothelial carcinoma, pT2-4 G3	92	18.50%
Inaguma 2016	27419370	Squamous cell carcinoma of the lung	53	15.10%
Inaguma 2016	27419370	Seminoma	71	14.10%
Inaguma 2016	27419370	Adrenal cortical carcinoma	31	12.90%
Inaguma 2016	27419370	Adenocarcinoma of the lung	52	7.70%
Inaguma 2016	27419370	Pleomorphic adenoma of the parotid gland	114	6.10%
Inaguma 2016	27419370	Papillary thyroid carcinoma	58	5.20%
Inaguma 2016	27419370	Thymoma	62	4.80%
Inaguma 2016	27419370	Hepatocellular carcinoma	92	1.10%
Inaguma 2016	27419370	Yolk sac tumor	3	0.00%
Inaguma 2016	27419370	Lobular carcinoma of the breast	58	0.00%
Stevers 2019	30171198	Mesothelioma, epithelioid	9	100.00%
Kaifi 2006	16400320	Gastrointestinal stromal tumor (GIST)	72	74.00%
Kaifi 2006	16400320	Leiomyoma	18	0.00%
Kaifi 2006	16400320	Leiomyosarcoma	11	0.00%
Kaifi 2006	16400320	Dermatofibrosarcoma protuberans	21	0.00%
Kim 2009	19239436	Lung, neuroendocrine tumor (NET)	55	61.80%
Blessmann 2012	22677742	Neurofibroma	23	100.00%
Blessmann 2012	22677742	Neurofibroma	20	100.00%
Blessmann 2012	22677742	Malignant peripheral nerve sheath tumor (MPNST)	17	41.00%
Thies 2002	12175686	Malignant melanoma	100	42.00%
